# Living Alone Among Older Adults in Canada and the U.S.

**DOI:** 10.3390/healthcare7020068

**Published:** 2019-05-07

**Authors:** Sharon M. Lee, Barry Edmonston

**Affiliations:** Department of Sociology and Population Research Group, University of Victoria, Victoria, BC V8W 3P5, Canada; be@uvic.ca

**Keywords:** living alone, older adults, older immigrants, Canada, U.S., older age-friendly housing

## Abstract

Increasing proportions of people, including older adults, live alone. Studying living arrangements of the elderly is important because these affect and reflect general well-being of the elderly and inform communities’ response to elderly housing needs. We analyze data from the 2006 Canadian Census and the 2006 American Community Survey to examine living alone among non-married older adults aged 55 and older in Canada and the U.S. The paper has two parts. First, we compare native- and foreign-born elderly to see if immigrants are less likely to live alone. Second, we examine factors associated with living alone among older immigrants. While older immigrants in both countries are less likely to live alone, the large differences are substantially reduced once various explanatory variables are considered. Comparisons of four gender/country groups of older immigrants show the positive role of economic and acculturation factors on living alone among older immigrants. With few exceptions, predictors of living alone are similar for older immigrants in Canada and the U.S.: living alone is mainly explained by a combination of economic and acculturation factors, taking demographic variables into account. Findings underline the need for age-friendly housing with innovative design and technology that can accommodate older people who live alone, including older immigrants who may have different needs and cultural preferences.

## 1. Introduction

Studying the living arrangements of older or elderly populations is important for several reasons. First, living arrangements affect and reflect family type and household structure among older people. These are in turn related to social support, inter-generational relations, health status, social isolation, satisfaction with life, and general wellbeing [1,2,3,4,5]. An older person living alone has different family and social relations from another living with a spouse or partner, or co-residing in a multi-generational family with an adult son or daughter and grandchildren, or co-residing with non-relatives. 

Second, another reason for studying elderly living arrangements pertain to the idea of “age-friendly communities” that incorporate suitable physical environments, including housing, transportation services, and home modification programs with innovative assistive technology and designs, with a supportive social environment that promotes positive social relations for older residents [2,6]. Older adults may not need as much space as that provided by the usual single-family home, so smaller housing units would be more appropriate. Declines in physical mobility and health may mean that older adults are less able to climb stairs, for example, or bend low or reach high to access shelves, kitchen cabinets, and other storage areas. Housing designs that minimize such potential barriers, and innovative technologies, for example, voice recognition software for operating doors and appliances, can be part of age-friendly communities. 

Factors that influence living arrangements of the elderly include preferences and resources that people have, and health and other constraints as they age. Residential options for older people include living alone if not married or partnered; living with spouse or partner only if married or partnered; co-residence with family members or extended family living; co-residence with non-family members; and institutional living, including retirement homes and assisted living facilities. Researchers often refer to the first two types of living arrangements (that is, living alone if not married or partnered, and living with spouse or partner only if married or partnered) as residential independence or independent living arrangements [7,8,9]. In the following review, the majority of references will be Canadian or U.S.-based, given the paper’s focus. 

### 1.1. Rise in Independent Living Arrangements among the Elderly

There has been a rise in independent living arrangements, as defined above, among older people in many countries, particularly in the west and more developed countries [10,11,12,13]. For example, a comprehensive report on living arrangements of older people around the world by the United Nations Population Division [13] shows marked differences between the elderly in more and less developed countries in independent living arrangements: 68 percent in the former and 20 percent in the latter live alone or with a spouse only. Conversely, 27 percent of elderly in more developed countries co-resided with a child or grandchild compared with 75 percent in less developed countries. Similar trends of independent living arrangements among the elderly are observed in Canada and the U.S. [7,8].

Over the past fifty years, there have been absolute and relative increases in the number of Canadian elderly people in independent living arrangements, mainly for married or partnered couples to live with spouse or partner only [1,5,14]. This contrasted with declining proportions in co-residential living arrangements, including living with other family members or with non-relatives. Recent data from the 2011 National Household Survey [15] show that among the population aged 65 and older, the majority (56.4 percent) lived as part of a couple and another 25 percent lived alone. In other words, over 80 percent of the population aged 65 and older were in independent living arrangements. The prevalence of living alone increases after age 50 for women and after age 70 for men, with a sharper increase for women [15]. 

Living arrangement patterns for the U.S. population aged 65 and older are fairly similar. Data for 2012 show that about 59 percent lived with a spouse or unmarried partner only and another 28.5 percent lived alone. Together, almost 88 percent of the population aged 65 and older were in independent living arrangements [16] (Table 3). The percent of older adults living alone was about 40 percent in 1990, but decreased to around 36 percent in 2000 and 2010 [17] (Table 72). As in Canada, living alone is higher among women, and increases with age, with sharper increases for women. For example, 47 percent of women aged 65 and older lived alone compared with 22 percent of men, in 2010 [17] (Table 72). 

#### 1.1.1. The Special Case of “Living Alone”

While living alone is not a new form of living arrangement, Klinenberg [10] describes the increased trend of “going solo” as a new “social experiment” that is fundamentally at odds with much of human history. Using the term “singleton” to refer to a person who lives alone, Klinenberg [10] documents a global increase in singletons, driven by increased economic prosperity and social security, “cult of the individual”, greater geographical mobility, greater job mobility, and several “revolutions”, specifically, in gender relations (leading to improved economic and social status of women), communications, mass urbanization, and longevity. 

The global rise in living alone occurs across the age range, but for this paper, we focus on “aging alone” [10], or the increase in older people living alone, a trend that is more pronounced in developed countries in the west and some parts of Asia [18]. Statistics on elderly living alone among developed countries include 38.4 percent in France, 32.7 percent in England and Wales, 30.8 percent in the U.S., and 22.5 percent in South Korea [18]. More elderly people are also living alone in China [19] and Japan [20]. For example, the 2015 census of Japan reported that the percent living alone among adults 65 and older was 12.5 percent among men and 20 percent among women [20]. Increased longevity is the main demographic reason for the rise in elderly people living alone. As people live longer, the risk of other life-course events increases, such as divorce and widowhood, which changes living arrangements, including a change to living alone [21]. As noted earlier, living alone is more frequent among elderly women [3,10,15,16,18,20] because of the gender gap in longevity and the common pattern of women marrying older men, which increases the risk of widowhood. 

Conventional beliefs about elderly people living alone have some truth. Many are widows, and experience poverty, social isolation, poorer physical and mental health, and lower life satisfaction and quality of life [1,3,4,10,21]. Turcotte and Schellenberg [22] report that poverty is highest among female seniors who live alone. However, despite the distinctive challenges of aging alone, many elderly people who live alone express a strong preference for this over other living arrangements, including living with adult offspring and grandchildren, if this option were available, preferring “intimacy at a distance” [10]. Some persons who live alone may be in a stable relationship with a partner who also lives alone. However, these “living apart together” (LAT) couples are more common among young adults. For example, only about 2 percent of people over 60 in Canada are in a LAT relationship [23]. Many elderly people who live alone value their independence and privacy, and would not willingly change their independent living arrangements, and especially fear losing their ability to reside independently [3,4,10]. 

#### 1.1.2. Older Immigrants

While statistics on living arrangements show an increased trend to independent living arrangements among the older population, several U.S. and Canadian studies show that immigrants, including elderly immigrants, are more likely to live in extended family living arrangements, and by implication, less likely to reside in independent living arrangements, including living alone [8,24,25,26]. The preference for extended living arrangements among immigrants has been explained by several factors, including economic factors (co-residence as an immigrant economic coping strategy) or cultural and acculturation factors (immigrants from some cultural backgrounds have stronger family values that encourage co-residence and less acculturated immigrants retain traditional customs including those about extended living arrangements). 

Still, older immigrants may be exposed to similar demographic forces such as increased longevity, gender gap in longevity, age gap between spouses, divorce and widowhood, as well as changing social norms and values regarding individualism, privacy, and independence, although the influence of these factors may vary between immigrant and native-born elderly. Older immigrants, particularly those who are more acculturated, may prefer independent living arrangements, including living alone if not married or partnered.

### 1.2. Research Questions and Contributions

This paper consists of two parts to addresses two research questions. First, are older immigrants less likely than Canadian- or U.S.-born elderly to live alone, once appropriate factors are considered? Second, we conduct additional analysis of older immigrants and examine the main factors associated with living alone among older immigrants in each country. Statistics and previous studies suggest that age and gender, and economic and acculturation factors will be particularly important. We examine similarities and differences in factors related to living alone among older immigrants across the two countries by comparing four groups by country and gender: Canada/female, Canada/male, U.S./female, and U.S./male. 

We recognize that population aging has become an important demographic trend in many parts of the world [13,27]. Many countries including European and Asian countries are responding to changing social, health, and housing needs with population aging [18,19,20]. We chose to compare the U.S. and Canada mainly because of our focus on older immigrants as these two countries have long histories of immigration and have relatively large immigrant populations (more details are provided in Section 1.3). 

This paper makes three contributions to existing research on living arrangements among the elderly. First, the focus on living alone highlights this form of living arrangement among older adults, with additional focus on immigrants. Much previous research on living arrangements of older immigrants had examined co-residence or extended living arrangements [24,25,26]. Living alone as a form of living arrangement has not received much attention. Although we do not directly examine the implications of living alone for older immigrants’ well-being, identifying the factors associated with living alone among elderly immigrants furthers our understanding of the demographic, economic, acculturation, and other characteristics of elderly immigrants who live alone. 

Second, this paper contributes to research on elderly immigrants in Canada and the U.S., a population which has not received much attention, as noted by Gelfand [28] and Wilmoth [9], compared to extensive research and discussion of the elderly in general. Aging immigrants are a growing part of the aging population in countries such as Canada and the U.S.; for example, 2006 Canadian census data show that more than one-quarter of the population aged 65 and older in Canada are foreign-born [22]. In the U.S., 12.7 percent of the population aged 55 and older are foreign-born [29]. 

Third, findings from this research provide evidence to support housing and other policy initiatives to respond to growing populations of older adults who may want to live alone but are unable to do so because of lack of appropriate housing designs and types. Most housing units in Canada and the U.S. are single-family units with front and/or backyards that become less appropriate over the lifecycle as children leave and parents age or become widowed. If we can document that more older adults are living alone (with potentially more who would have preferred to live alone but for lack of appropriate housing), public and private sectors can use this information to promote age-friendly housing and communities (for example, smaller housing units with close by amenities such as shops and healthcare facilities). The concept of “environmental gerontology” highlights the need for a multi-disciplinary approach to designing neighborhoods and housing that facilitate “mobility, independence, and quality of life of older people living in the community” [6] (116).

Finally, this study is the first comparative analysis of living alone among older immigrants in Canada and the U.S., offering a useful comparison of two large immigrant-receiving countries that have older immigrants from many different countries of origin. While Canada and the U.S. are distinctive in many ways, both countries have long histories of immigration with relatively high levels of current immigration from diverse source countries. In the following section, we discuss why a comparative study of Canada and the U.S. can be especially productive in advancing understanding of living alone among older immigrants.

### 1.3. Comparing Canada and the U.S.

While there are distinctive challenges in cross-country research, including the need for comparable variables and sensitivity to historical and contextual differences, a comparative analysis can advance knowledge in many unique ways. A comparative analysis has the advantage of allowing researchers to conduct similar analyses using different data from the countries selected to identify similarities and differences in how various factors and characteristics affect the outcome being examined. If the influence of particular factors is similar, this increases confidence in the validity and reliability of the findings.

Comparing Canada and the U.S. for this analysis is not simply based on the fact that they are North American neighbors with a long mostly amicable joint history, and that the two countries have been strong allies in modern times. Canada and the U.S. are far from “two peas in a pod” [30], but besides being North American neighbors and close economic trading and foreign policy partners, there are other similarities and differences between Canada and the U.S. that make for a fruitful comparative study of living alone among older adults, including immigrants. There are also differences, for example, Canada’s population and economy are much smaller than that of the U.S.: Canada’s population is about 33.5 million in 2011 [31] compared with 308.7 million in the U.S. in 2010 [32]. However, Canada and the U.S. share several sociodemographic trends. We describe similarities as well as differences to show the value of such a comparative study of living alone among older adults, including older immigrants.

#### 1.3.1. Role of Immigration

First, immigration has always been a major factor in both countries, historically and today. Canada and the U.S. are among the leading destinations for global migration streams in recent decades [33]. Both countries have long histories of immigration and generally take pride in their immigrant heritage. 

However, we should note differences in immigration policies and systems. Canada has a selective points-based immigration system whereby potential immigrants are screened based on such human capital characteristics as age, education, English and/or French language proficiency (Canada’s two official languages), and adaptability whereas the U.S. immigration system is primarily based on family reunification. 

While the U.S. continues to admit more immigrants than other major immigrant destination countries [34], immigration has a larger influence on Canada’s population. The percent foreign-born of Canada’s national population stands at 24 percent compared with 13 percent for the U.S. [34]. Immigration has been the main source of Canada’s population growth since 1993/1994 [31]. For the year ending June 30, 2012, net international migration accounted for two-thirds of Canada’s population growth [31]. Population growth increasingly stems from the contribution of immigration because fertility levels are below replacement. In contrast, immigration accounts for a lower percent of U.S. population growth, at about one-third of U.S. population growth in recent decades [34].

#### 1.3.2. Population Aging

Second, population aging is another demographic trend shared by both countries. The Canadian population is aging, indicated by increased median age of the population from 26.2 in 1971 to 40.0 in 2011 [31]. Elderly immigrants are a growing segment of Canada’s aging population, with more than one-quarter of the population aged 65 and older being foreign-born [22]. 

Similar population aging trends are observed in the U.S. [35]. The median age of the U.S. population has steadily increased, from 30.0 in 1980 to 37.2 in 2010 [17] (Table 7). The percent of the U.S. population aged 55 and older has also increased, from 20.8 percent in 1980 to 24.9 percent in 2010 [17] (Table 7). Of the population aged 55 and older, 12.7 percent are foreign-born [29]. 

Aging-in-place of younger foreign-born cohorts and the immigration of older immigrants contribute to the growth of the elderly immigrant population in each country. While both the Canadian and U.S. populations are aging, and aging immigrants are part of this demographic trend, statistics cited above show that Canada’s population is older and elderly immigrants are a larger proportion of its elderly population. 

#### 1.3.3. Racial and Ethnic Diversity

Third, closely related to the role of immigration is the expanded racial, ethnic, and cultural diversity of the Canadian and U.S. populations. Mainly because of immigration from Asia and other non-traditional (that is, non-European or North American) sources in recent decades, Canada’s population has evolved from one dominated by the two founding peoples (British and French) and the indigenous (Aboriginal) population to the current situation where over two hundred ethnic origins were reported, and thirteen different ethnic origins had one million or more responses [36]. 

In 2011, close to 20 percent (19.1 percent) of Canada’s population identified as members of “visible minority” groups, that is, racial minority groups other than Aboriginal peoples. The *Employment Equity Act* of Canada defines visible minorities as ‘persons, other than Aboriginal persons, who are non-Caucasian in race or non-white in colour’. The visible minority population consists mainly of the following groups: South Asian, Chinese, Black, Filipino, Latin American, Arab, Southeast Asian, Korean, and Japanese [36].

The U.S. population has also been transformed by increased racial and ethnic diversity, also closely related to immigration. The main group, non-Hispanic White, has been slowly declining as a percent of the total population, to 63.7 percent in 2010, down from 69.1 percent in 2000. This means that other racial groups and Hispanics have been increasing in numbers and proportions, and together comprise about 36 percent of the total U.S. population in 2010 [37]. 

Asians were the fastest growing racial minority between 2000 and 2010, but account for just 5.6 percent of the population in 2010. The most notable change has been the growth of the Hispanic or Latino population to become the largest minority population in the U.S. since the 1990s. Hispanics are now 16.3 percent of the U.S. population compared with 13.6 percent Black and 5.6 percent Asian, the other main racial minority groups [37]. 

While both Canada and the U.S. have become more racial and ethnically diverse, there are important differences, including the larger share of racial/ethnic minority populations in the U.S. (36 percent), compared with 19 percent of visible minority groups in Canada, and the large presence of Hispanics in the U.S. 

## 2. Data and Methods

We analyze data from two data sets. Data for Canada are from the Public Use Microdata File (PUMF) on individuals in the 2006 Census of Canada (see [38] for detailed technical and data documentation). These data are a 2.7 percent representative sample of the population enumerated in the census. The microdata sample for individuals is selected using a three-phase sampling plan. The first sampling phase consists of the sample of one-fifth of the population (20% sample data). This is a cluster sample. It consists of all households who completed the long questionnaire in the census. This sample was divided into two parts that are representative of Canada in order to create two sampling frames used to select the microdata samples. The first frame was used to select microdata from the individuals file. The second frame was used to select microdata from the hierarchical file. The third phase consisted in selecting records from the individuals file. The final sample contains 844,476 records, representing 2.7% of the target universe, which is the Canadian population.

U.S. data are from the 2006 American Community Survey (ACS) Public Use Microdata Sample (PUMS) (see [39,40] for detailed information on the ACS and technical documentation for the 2006 ACS PUMS file). During previous decennial censuses up to the 2000 census, most households received a short-form questionnaire, while one household in six received a long form that contained additional questions and provided more detailed socioeconomic information about the population (this was the long-form census). The 2010 census was a reengineered short-form only census, counting all residents living in the United States and asked for name, sex, age, date of birth, race, ethnicity, relationship and housing tenure, taking just minutes to complete. The more detailed socioeconomic information once collected via the long-form questionnaire is now collected by the ACS. The 2006 ACS PUMS consists of 1,277,561 housing unit records (1 percent of all housing units) from which 2,923,336 person records were sampled. 

We chose these two data sets because in 2005, the United Nations published a pioneering piece on living arrangements of older people around the world [13] and in the following year, the World Health Organization released its guide to global age-friendly cities in response to global population aging [27]. This motivated us to conduct a comparative analysis of living arrangements of the elderly in Canada and the U.S. (see previous Section 1.3 for why we focus on Canada and the U.S.), using comparable nationally representative data from each country from around the time of the U.N. and WHO publications to provide baseline findings for future research on the subject. 

For statistical analysis, we define the study sample as persons 55 years and older. The meaning and definition of “aging” and the “elderly” are increasingly open to question. Researchers studying the “elderly” or the “aged” recognize that using a particular age to define the elderly is arbitrary. We recognize that the “elderly” are a very heterogeneous group and reaching a particular age (be it 55, 60, 65 or 70) does not always imply declining economic or health status. Many statistics on the elderly refer to persons aged 65 and older, or persons aged 55 and older. In this study, we use age 55 in order to show more clearly potential differences that occur between age groups and to cover a wider age range at the “older” ages. 

Given the outcome variable—living alone—we exclude persons who are married or living common-law (the latter status is officially recognized in Canada and often treated as equivalent to being married) or co-habiting. We include men and women, who are never married, separated, divorced, or widowed. We exclude older persons living in group quarters. Recent statistics show that for the population aged 65 and older, over 92 percent in Canada live in private households [41], and the comparable figure is 95 percent in the U.S. [42] (Table 35). For our study population of persons aged 55 and older, the percentages would likely be higher.

We identify Canadian-born or U.S.-born elderly and immigrants from responses to the questions on citizenship at birth and place of birth. Persons who are Canadian or U.S. citizens at birth are considered native-born while persons who are not Canadian or U.S. citizens at birth are considered immigrants. This avoids including persons born abroad to Canadian or U.S. citizens as immigrants (based on foreign place of birth) as these persons are not considered immigrants in Canada or the U.S., respectively.

Variables included in the analysis are as directly comparable as possible across the two data sets. We note where it is not possible to develop directly comparable categories for some variables.

### 2.1. Outcome Variable

The outcome variable, living alone, is coded as a binary variable (1 = live alone; 0 = don’t live alone) based on responses to questions on household type, family structure, and individual family status.

### 2.2. Explanatory Variables and Expected Effects

We include explanatory variables that previous research had shown to influence living arrangements of older adults. Expected results are based on previous research and published statistics. 

#### 2.2.1. Demographic Variables

Age is coded in seven age groups, 55–59, 60–64, 65–69, 70–74, 75–79, 80–84, and 85 years or older for descriptive analysis. We use 5-year age groups because 2006 Canadian census public-use microdata are limited to five-year age groups. Age is recoded into a continuous variable in the multivariate analyses. We use the mid-point of each 5-year age category to assign age values for the Canadian sample (for example, persons aged 55–59 were assigned an age of 57.5).

Statistics on living alone from Statistics Canada [41] and the U.S. Census Bureau [42] suggest that living alone increases with age but these statistics do not take into account marital status, health, and other factors that may make it more difficult for older adults to live alone. It is possible that living alone increases with age, but it is equally likely that once additional factors are considered, age may have negative or only modest influence on living alone.

Gender is a binary variable (0 = female; 1 = male). Living alone is expected to be more likely among women because of women’s longer longevity. However, this may not be the case once other factors are considered.

Marital status includes four non-married categories: divorced, separated, widowed, and never-married. Living alone may be more likely among widowed adults.

#### 2.2.2. Economic Variables

The influence of economic variables is expected to be positive, as living alone requires adequate income to pay for a housing unit that is inhabited by only one person. 

Education is coded in five categories: less than high school, high school graduate, some post-high school education but less than a Bachelor’s degree, Bachelor’s degree, and post-Bachelor’s degree. Education is expected to have a positive influence on living alone.

Individual income is coded in six categories: less than $10,000; $10,000–19,999; $20,000–39,999; $40,000–59,999; $60,000–99,999; $100,000 and over for descriptive analysis. Income and other monetary variables are measured using Canadian dollars in the Canadian sample and U.S. dollars in the U.S. sample. In 2006, the Bank of Canada exchange rate was around US $1 equals CAD 1.14.

In the multivariate analyses, individual income is a continuous variable. Living alone is expected to increase with income.

Government retirement income is a binary variable (0 = received less than $100 in government retirement income during the past year or 1 = received $100 or more in government retirement income during the past year). In Canada, government retirement income refers to benefits from the Canada or Quebec Pension Plan. In the U.S., government retirement income refers to payments and benefits from the Social Security Administration. Having government retirement income increases the likelihood of living alone.

Guaranteed retirement income is a binary variable (0 = received less than $100 in retirement income from a private or personal pension plan during the past year or 1 = received $100 or more in retirement income from a private or personal pension plan during the past year). Guaranteed retirement income refers to regular income received from being a member of an employer’s pension plan, payments from individual annuities, private pensions paid to widows or widowers, pensions of retired civil servants, and other annuities paid to individuals by a private insurance company. Having guaranteed retirement income is expected to increase the chances of living alone.

Homeownership is a binary variable (0 = does not own home or 1 = own home). Homeownership’s influence is expected to be positive on living alone because owning a home implies having sufficient economic resources to own a home. In addition, homeownership facilitates living alone, removing the need to look for alternative housing in the event of marital dissolution or widowhood.

#### 2.2.3. Cultural and Acculturation Variables

Culture and acculturation are closely related but distinct concepts. Ethnic origin, language background, religion, and other characteristics are usually used to indicate cultural background. Given differences across ethnic groups on other characteristics such as marriage, fertility, and family patterns, ethnicity has frequently been used as a sociodemographic variable to indirectly measure these differences. We describe ethnic origin as a demographic variable in the descriptive analysis. For immigrants, acculturation is usually indicated by duration of residence in the host country and proficiency in host country language [24,43,44]. 

Ethnic origin is coded using fifteen groups. These are “American” (in the U.S. sample) or “Canadian” (in the Canadian sample); British; French; Other European; Arab or Middle Eastern; South Asian/Asian Indian; Chinese; Filipino; Korean; Vietnamese; Other Asian; Latin American/Latino/Hispanic; African, Black, or Caribbean; Other single ethnic origins (including persons reporting Aboriginal only in the Canadian sample and Native American or Alaskan Native or Native Hawaiian/Pacific Islander only in the U.S. sample); and Multiple ethnic origins. Persons reporting ethnic origins that are culturally closer to mainstream American or Canadian culture (that is, American, Canadian, and various European groups) are more likely to live alone.

For immigrants, knowledge of official languages (in Canada) or proficiency in English (in the U.S.) is coded using four categories. Besides being an indirect indicator of acculturation, language knowledge or proficiency implies an ability to communicate and navigate social and other situations and understanding of broader societal norms.

The four categories range from excellent to poor competence in Canada’s two official languages (English and French) or in English (for the U.S. sample), although the specific definitions differ for Canada and the U.S. For the Canadian sample, the four categories are (1) English or French mother tongue, and English or French home language; (2) other mother tongue, and English or French home language; (3) other mother tongue, and other home language, knows English or French; and (4) other mother tongue, other home language, and does not know English or French. For the U.S. sample, the four categories are: (1) only speaks English; (2) speaks English very well; (3) speaks English well or not well; and (4) does not speak English. Cultural closeness to the host country and/or acculturation decreases from category 1 to 4 for both the Canadian and U.S. samples, and living alone is expected to decrease from the first to the fourth category of the language variable. 

Duration of residence for immigrants measures how many years immigrants have resided in Canada or the U.S. It is coded in five categories for descriptive analysis, from 0–9, 10–19, 20–29, 30–39, and 40 years or more. In the multivariate analyses, duration of residence is a continuous variable. Duration of residence is expected to have a positive influence on living alone as increased duration implies greater acculturation.

#### 2.2.4. Other Control Variables

Place of residence indicates metropolitan and non-metropolitan residence, and residence in several specific Canadian and U.S. cities. Metropolitan categories for this variable include three Canadian and five U.S. cities with the largest number of older immigrants. We include only three Canadian cities because immigrants in Canada are highly concentrated in them: 63.4 percent of Canada’s immigrants reside in these three cities [36].

The codes for place of residence are (1) Montreal (Canada) or Chicago (U.S.); (2) Toronto (Canada) or Los Angeles (U.S.); (3) Vancouver (Canada) or Miami (U.S.); (4) New York City; (5) San Francisco; (6) other metropolitan areas; and (7) non-metropolitan areas. Living alone is expected to be higher in non-metropolitan areas because of lower cost of housing which facilitates independent living arrangements, including living alone.

### 2.3. Methods of Analysis

We begin with descriptive analyses to describe and compare the study samples. For multivariate analyses, we use Stata 12 statistical software [45] to estimate several logistic regression models because the outcome variable is coded as a binary variable. For the first research question on nativity differences in living alone, we estimate two regression models (Models I and II). Model I is estimated separately for Canada and the U.S., for all non-married elderly, aged 55 and older. Each equation includes dummy variables for nativity and gender, and other explanatory variables described above (except for duration of residence because it is collinear with nativity). Second, we estimate a logistic regression of living alone for all non-married elderly, aged 55 and older (Model II), for four groups: females in Canada, males in Canada, females in U.S., and males in U.S. Each equation includes a dummy variable for nativity, and explanatory variables described above (except for duration of residence because it is collinear with nativity, and gender). Results from Models 1 and 2 address our first research question. 

For the second research question on predictors of living alone among older immigrants, we limit analysis to older immigrants only, and estimate a logistic regression model of living alone (Model III) for four groups: females in Canada, males in Canada, females in U.S., and males in U.S., to identify and compare predictors of living alone among older immigrants. Model III includes duration of residence, in addition to other explanatory variables.

For interpreting the logistic regression results, we calculate predicted probabilities for each explanatory variable using the margins command in Stata 12 [45]. The predicted probabilities provide a useful interpretation of the net effect of each categorical variable on living alone, evaluated by holding constant the effects of all other variables in the model [46]. Multiplying predicted probabilities by 100 converts them into percentages or proportions, which facilitates presentation and discussion of results. We include the logistic regression results from which the predicted probabilities are calculated in the Supplementary Tables.

## 3. Results

### 3.1. Descriptive Results

Selected characteristics of the Canadian and U.S. study samples are shown in Table 1. Non-married immigrant elderly in both Canada and the U.S. are much less likely to live alone than native-born elderly. In Canada, 54.8 percent of non-married older immigrants live alone, compared with 70.7 percent of Canadian-born non-married elderly (a difference of 15.9 percent). In the U.S., 51.7 percent of non-married older immigrants live alone, compared with 73.2 percent of U.S.-born non-married elderly (a difference of 21.5 percent). The gap is larger in the U.S.

#### 3.1.1. Demographic Characteristics

Gender: There are more females in both samples, about 69 percent. There are also more women in the immigrant samples, at around 73–74 percent in both the Canadian and U.S. samples. 

Age: The distribution across age categories is as expected, with higher percents in the younger age categories. The U.S. immigrant sample has higher percents in the younger age categories. 

Marital Status: Marital status refers to non-married categories only. Being widowed is the most common marital status for both Canadian and U.S. samples, with a higher percent widowed among immigrants in Canada. The percent divorced is higher among the native-born in both samples. 

Ethnic Origin: For describing the sample, ethnic origin is considered a demographic characteristic. Most native-born Canadian and U.S. elderly report European or multiple origins but immigrants are distributed over a wider range of ethnic origins compared with the native-born. There are two striking differences between the Canadian and U.S. immigrant samples. First, older immigrants in Canada have higher percentages of people reporting European origins (72.8 percent—this percent includes the 25 percent reporting multiple origins), compared with 33.8 percent (including the 5.9 percent reporting multiple origins) in the U.S. sample. Studies of ethnic origin in Canada show that persons reporting multiple origins are mainly reporting “Canadian” in combination with other European origins, and persons reporting “Canadian” used to report European origins, particularly French or British [47,48,49]. Second, almost one-third (32.1 percent) of older immigrants in the U.S. report Latin American/Hispanic/Latino origin while no single ethnic group dominates the immigrant sample in Canada (the largest three are British at 13.6 percent, Chinese at 8.7 percent, and South Asian at 6.2 percent). 

#### 3.1.2. Economic Characteristics

Education: On average, older immigrants in Canada have more years of schooling compared to Canadian-born elderly (a mean of 12.1 years versus 11.5 years) whereas in the U.S., older immigrants have fewer years of schooling, with a mean of 10.3 years versus 12.3 years for the U.S.-born. The distribution across levels of educational attainment of Canadian-born and immigrant elderly is generally quite similar but higher percents of immigrants in the U.S. sample are in the lower educational categories. 

Income: In both samples, older immigrants have lower mean incomes, and the percent of immigrants in the two lowest income categories exceeds that of the native-born in both countries. Homeownership: Homeownership is higher for the U.S. sample (70 percent are homeowners compared with 62 percent for the Canadian sample). However, immigrants in Canada are more likely to own their homes (67 percent, compared with 60 percent for Canadian-born elderly), while immigrants in the U.S. sample are less likely to be homeowners (62 percent, compared with 71 percent of U.S.-born elderly).

#### 3.1.3. Other Characteristics

Metropolitan Residence: Notably higher percentages of elderly immigrants in both samples reside in metropolitan areas. The metropolitan concentration of older immigrants in the U.S. is higher, at 94 percent, compared with 86 percent in Canada. U.S.-born elderly are also more likely to reside in metropolitan areas (73 percent) compared with Canadian-born elderly (at 59 percent).

#### 3.1.4. Immigrant-Specific Characteristics

There are two immigrant-specific characteristics in Table 1: duration of residence in the host country and host country language proficiency. 

Duration of Residence: On average, older immigrants in Canada have resided in Canada for 37.7 years compared with 34.8 years for older immigrants in the U.S. Higher percentages of elderly immigrants in the Canadian sample have resided in Canada for 40 or more years (43.8 percent) compared with 36.4 percent of immigrants in the U.S. sample. More immigrants in the U.S. sample are recent arrivals: 10.7 percent have been in the U.S. for less than 10 years, compared with 5.5 percent of immigrants in the Canadian sample. 

Language Proficiency: As noted earlier in the section describing variables and in Table 1, categories of the language proficiency variable are not directly comparable between the two samples. However, there is a similar pattern for interpreting its effects, that is, acculturation (indirectly indicated by language proficiency/knowledge) decreases from category 1 to category 4. About two-thirds of the Canadian sample are in the first two categories and would be considered highly acculturated but 14.6 percent are in the fourth category (considered the least acculturated). 44.7 percent of the U.S. sample are in the first two categories while 19 percent are in the fourth category. 

### 3.2. Logistic Regression Results

Logistic regression results from Models I and II focus on the role of nativity on living alone. We begin with results from Model I, which was estimated separately for Canada and the U.S. Complete logistic regression results for Model I are in Supplementary Tables S1A and S1B. Table 2 compares observed (or descriptive) and adjusted results by nativity. Given the large amount of statistical results, we do not show predicted probabilities for all the explanatory variables included. The complete tables of predicted probabilities are available upon request. We highlight differences by nativity, as this is the main focus for this part of the analysis. 

While the role of nativity is statistically significant (foreign-born older adults are less likely to live alone), the observed large gaps in living alone between native- and foreign-born elderly are substantially reduced once other factors in the equation are taken into account. Specifically, the observed difference of 15.9 percent between Canadian-born and immigrant elderly living alone is reduced to 3.4 percent, and the observed difference of 21.5 percent between U.S.-born and immigrant elderly is reduced to 1.6 percent. This shows that differences in living alone between older native-born and immigrants are modest, once all other factors in Model I are considered.

Results for Model II, estimated for four groups, Canada/female, Canada/male, U.S./female, and U.S./male, are shown in Table 3. Again, we highlight the role of nativity. Complete results from the logistic regressions for Model II are shown in Supplementary Tables S2A–D. While the influence of nativity remains statistically significant (except in the logistic regression for the U.S. female sample—Supplementary Table S2C), fairly large observed differences in living alone between native- and foreign-born females and males in Canada and the U.S. are greatly reduced. 

**Figure 1:**
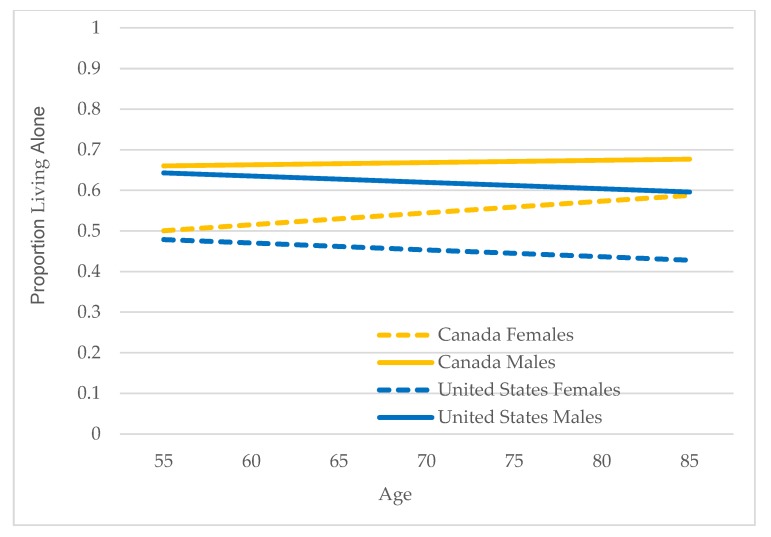
Predicted proportions living alone by age: Canada and U.S., female and male older immigrants.

**Figure 2:**
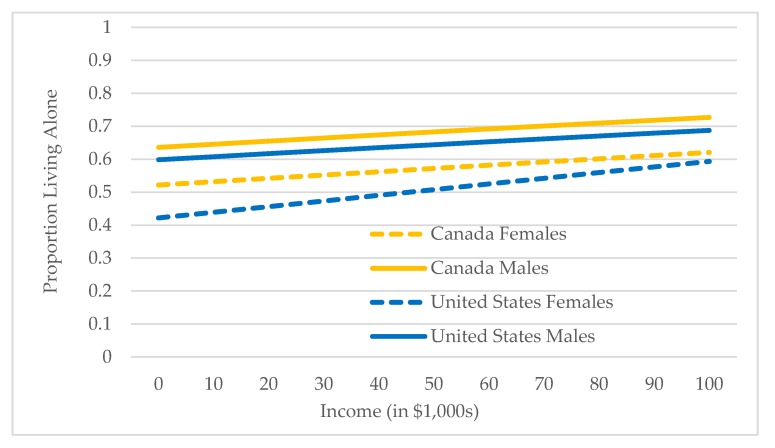
Predicted proportions living alone by individual income: Canada and U.S., female and male older immigrants.

**Figure 3:**
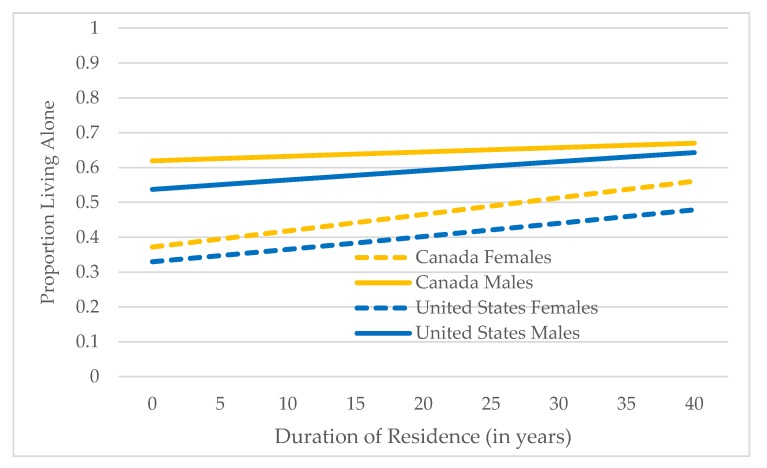
Predicted Proportions Living Alone by Duration of Residence: Canada and U.S., Female and Male Older Immigrants.

Model III is estimated for older immigrants only and addresses the second research question: what factors are associated with living alone among older immigrants? Complete results from estimating Model III for Canada/female, Canada/male, U.S./female, and U.S./male are shown in Supplementary Tables S3A–D. Table 4 shows predicted probabilities for categorical variables from estimating Model III for the four gender/country groups and results for three continuous variables—age, individual income, and duration of residence—are shown in Figure 1, Figure 2 and Figure 3 (in the figures, predicted probabilities have been converted to proportions to facilitate presentation and description). 

#### 3.2.1. Demographic Characteristics

Age: Age is an important factor in living arrangements among older adults because of age-related health declines which can be expected to influence the ability to live alone [7,11,18]. The results for age are shown in Figure 1. Age differences are statistically significant for female immigrants in both Canada and the U.S., but not for males. Living alone increases with age for older immigrants in Canada, with sharper increases for females. The influence of age is negative for both sexes in the U.S., is more pronounced for females, and as noted, not statistically significant for males. 

Marital Status: The results for marital status are shown in Panel A, Table 4. We highlight the role of marital status because of conventional views that most elderly who live alone are female widows. Logistic regression results show that the influence of marital status were not statistically significant for most groups and differences in predicted probabilities of living alone are relatively modest. Around 54–57 percent of female immigrants in Canada live alone across different marital status categories. Among male immigrants in Canada, widowed males have the lowest proportion living alone while separated males have the highest proportion. Differences by marital status are larger than those for females but are still modest.

Among female immigrants in the U.S., those who are separated are least likely to live alone while divorced female immigrants are most likely to live alone. Among males, widowed males have the lowest proportion living alone, while divorced and never-married males have the highest proportions living alone. In both samples, divorced older immigrants have higher proportions living alone. 

#### 3.2.2. Economic Factors

Individual income: Individual income results are shown in Figure 2.

As expected, the proportions living alone increase with income, with the sharpest increase observed for females in the U.S. The influence of other economic factors are shown in Panel B, Table 4, and are all also in the expected positive direction.

Government and private retirement income: Older immigrants who have government or private retirement income are more likely to live alone. These results hold across all groups, but the impacts are larger for males in both Canada and the U.S.

Home ownership: Older immigrants who are homeowners are more likely to live alone, a pattern observed for all four gender/country groups. Older male immigrants in Canada who are homeowners have the highest proportion living alone.

Educational Attainment: The proportions living alone increase for all four groups as educational attainment increases. The proportions living alone are higher among males in both countries at each level of educational attainment.

#### 3.2.3. Acculturation Factors

The results for ethnic origin and language proficiency are shown in Panel C, Table 4.

Ethnic Origin: The influence of ethnic origin is not statistically significant for almost all ethnic origins. We interpret the influence of ethnic origin as a largely cultural and acculturation variable, but acknowledge that ethnic origin relates to other characteristics that can also influence living alone, such as ethnic group differences in marriage and fertility patterns, which relate to family size and availability of family for elderly co-residence. The lack of statistically significant results for all but a few ethnic origins suggests that this may not be an adequate proxy for characteristics that affect living alone among older immigrants.

However, ethnic group differences in living alone do generally support cultural expectations: older immigrants of European backgrounds are culturally closer to “mainstream” Canadian and U.S. culture, and higher proportions of these groups live alone. In contrast, lower proportions of older immigrants reporting Asian, Latin American, and other non-European origins live alone. Older female immigrants of all ethnic origins in both Canada and the U.S. are less likely to live alone than male co-ethnics (there are two exceptions to this pattern: Korean and Latin American female immigrants in Canada have higher proportions who live alone compared to co-ethnic males). 

Language Proficiency: This variable indicates high to low linguistic and related acculturation (from Category 1 to Category 4) and its influence is as expected. The proportions living alone decrease from Category 1 to Category 4 for both samples and for both genders. For example, 60 percent of older female immigrants in Canada in Category 1 live alone, compared with 43 percent of female immigrants coded Category 4. The difference by linguistic acculturation is larger among males: 72 percent of male immigrants in Canada classified in Category 1 live alone versus 53 percent of males classified in Category 4. 

Duration of Residence: Acculturation is also indicated by duration of residence, increasing as years of residence increase. As expected, living alone among older immigrants increases with duration of residence, shown in Figure 3. 

The increase is sharper among female immigrants in both Canada and the U.S., and immigrants in Canada (both females and males) have higher proportions living alone compared to their U.S. counterparts at all values of duration of residence. 

#### 3.2.4. Other Controls

Place of Residence: Older immigrants who live in non-metropolitan areas are more likely to live alone, a pattern that is similar for all four sub-groups. Lower proportions of older immigrants in Canada who reside in one of Canada’s three largest immigrant destination cities (Montreal, Toronto, and Vancouver) live alone, compared to immigrants who live in other metropolitan and non-metropolitan areas. For older immigrants in the U.S., a similar pattern holds, except for those who reside in Miami where the proportion living alone (69 percent) is quite close to the percent living alone in non-metropolitan areas. 

## 4. Discussion

We return to our two research questions in this section. The first question focuses on the role of nativity and asks whether non-married older immigrants are less likely than Canadian- or U.S.-born non-married elderly to live alone. Descriptive results show that older immigrants in Canada and the U.S. are much less likely to live alone than native-born elderly. The difference is larger in the U.S. This finding is consistent with other research showing lower rates of independent living arrangements, including living alone, among immigrants, including older immigrants [9,24,43]. 

However, once appropriate factors are taken into account, nativity differences, while still statistically significant, are substantially reduced. Differences by gender and nativity are also reduced or become modest once various factors are considered. These findings suggest that aggregate differences in living arrangements between older immigrants and native-born elderly are largely due to differences in demographic, economic, and acculturation characteristics between the older native-born and immigrant populations. Findings show that notable proportions of older adults, including immigrants, in both countries live alone, reinforcing the need to consider these groups when discussing age-friendly communities.

Our second research question is directed at older immigrants and examines predictors of living alone among non-married older immigrants in Canada and the U.S. The main findings show higher levels of living alone for older male immigrants in both Canada and the U.S., across different characteristics, including age, marital status, income, education, and duration of residence. With some exceptions, the proportion living alone is higher among immigrants in Canada across different characteristics. 

Factors influencing living alone are generally similar for older immigrants in Canada and the U.S., suggesting that living alone among older immigrants is mainly explained by a combination of economic and acculturation factors, after taking demographic variables into account. More acculturated older immigrants, and immigrants with more economic resources, are more likely to live alone, findings that are consistent with previous studies on extended living arrangements among older immigrants: the predictors of living alone are opposite to those for extended living arrangements where less acculturated older immigrants with fewer economic resources are more likely to co-reside [26,50].

Aggregate statistics on older people living alone contribute to widespread beliefs and images that older women are more likely to live alone. It is of course correct that higher proportions of older women live alone, as shown in statistics from many countries [13,18,20], as well as Canada and the U.S. However, when appropriate demographic, economic, and acculturation factors are taken into account, this study of older immigrants shows that male older immigrants are more likely to live alone. Therefore, being male is a stronger predictor of living alone among older immigrants, once additional appropriate factors are considered. Perhaps the inclusion of several key factors in this analysis such as acculturation measures (host language proficiency and duration of residence) and economic resources (indicated by not just individual income but access to private and government pensions) permitted a more comprehensive examination of the role of gender on living alone among older immigrants. Other factors such as stronger male preference for living alone and greater social acceptability of males living alone could also be implicated. Different research using different data with information on availability and type of kin, social networks and relationships, and gender differences in preference for and acceptability of living alone would be useful to further explore these findings.

Another widespread image of elderly people who live alone is that of elderly widows living alone. Again, this is not entirely wrong, given women’s longer longevity and the common age gap between spouses. However, once appropriate factors are taken into account, older immigrants who are widowed are not the most likely group to live alone, compared to other marital status groups. It is possible that widowed older immigrants have adult children with whom they can co-reside following widowhood, an option that may be unavailable to divorced, separated, and never-married older immigrants. Divorced and separated older immigrants are more likely to live alone than the widowed, and in the U.S., older male immigrants who are never-married are as likely as divorced males to live alone. Marital disruptions due to divorce is therefore a better predictor of living alone among older immigrants than widowhood. Marital disruptions can also be associated with other forms of disruptions such as moving away, which also disrupts previous family and social networks, thereby increasing the chances of living alone. Unfortunately, we are unable to examine the role of geographical mobility as well as other factors such as gendered differences in cultural norms about living alone and subjective preference for living alone with the data examined in this paper. 

This paper contributes to the literature on living alone and housing in two ways. First, we show that notable percentages of older adults, including older immigrants, in two large countries with aging populations, live alone. This trend is expected to continue and reinforces the need for more private and public policies to design and build age-friendly communities that allow older adults to continue to live independently and participate fully in their community. Second, the findings show that once appropriate factors are taken into account, there are only modest differences between native-born and immigrant elderly’s likelihood of living alone. This suggests that elderly immigrants should be included in housing initiatives that include more units geared towards elderly living alone, instead of mistakenly assuming, based on aggregate statistics, that elderly immigrants are somehow different, and are less likely to live alone and do not need to be included in these efforts.

Many countries have already implemented initiatives on age-friendly communities in response to population aging and the rise in independent living arrangements among the elderly, including living alone [6,18,20]. Such initiatives would need to include community services such as home care services, senior community centers, transport services, housing designs such as greater availability of smaller housing units, for example, one or two-bedroom single-level apartments, and other factors such as support for innovative technology that may make it easier for non-married older adults to continue to live alone at older ages. 

Additionally, older immigrants may have different cultural preferences in housing design and use of technology. For example, Chinese immigrants may place great importance on the role of feng shui in housing alignment and design, immigrants may be less familiar with advanced technology, and some immigrants’ accented speech may pose challenges for voice-recognition software. These potential differences would have to be considered in elderly housing designs and use of technology in planning age-friendly housing. 

While this study has produced some new and useful findings, we note several limitations. First, this is a cross-sectional analysis, and findings refer only to the period when the data were collected in 2006. We do not know if the living arrangement recorded at time of data collection is temporary or permanent, and the findings cannot speak to trends in factors related to living alone. 

Second, the sampling frame for both data sets are private households and individuals and families in private households. This misses the population in group or institutional living quarters, an important limitation for studying the elderly. As health declines accelerate with increased age, the oldest old are less likely to live alone in private households and more likely to be in group housing such as retirement or assisted living housing. This limitation may be implicated in the finding of age’s negative influence on living alone among U.S. female older immigrants. 

Third, the outcome, living alone, poses some conceptual challenges. Living alone is one type of living arrangement, and living arrangements are inherently dynamic and may be recursive. This means that an individual can transit through different types of living arrangements over her/his life (for example, living at home with parents → living alone as a young adult → living with spouse upon marriage → living with spouse and children → living alone upon divorce → remarriage, living with new spouse → widowed, living alone). In this example, living alone occurs at different stages over the life course, and has different determinants and implications. The study of living arrangements has therefore to be particularly sensitive to age, gender, and life course influences, including marital status. 

Fourth, there are measurement challenges for studying living alone as a form of living arrangement. The data examined in this study do not tell us whether the person living alone is in a relationship with another person (the “living apart together” couples noted earlier). It is likely that the predictors and implications of living alone for such individuals would differ in important ways from others who live alone and are not in a relationship.

Finally, while the census and ACS data used are appropriate for identifying and comparing sociodemographic, economic, and acculturation factors on living alone among older immigrants, there is no information on other factors that influence older immigrants’ living arrangements, including the key role of health status (the ACS includes a question on disability but there is no comparable information in the Canadian census). Other unmeasured factors include availability of family or friends to share housing, and community characteristics that either facilitate or discourage living alone (for example, community support for innovative housing designs and technology, and availability and affordability of housing units for older singletons).

## 5. Conclusions

We began our analysis by making no assumptions about whether living alone is the “best” living arrangement for non-married older adults. The increased social trend to elderly residential independence suggests that most elderly prefer independent living arrangements [10], but we recognize that for some older adults, particularly immigrants, co-residence may actually be preferable and more advantageous, and lowers the risk of social isolation [1,4,9]. 

However, as we reflect on our findings, a picture emerged suggesting that living alone is associated with characteristics that can only be described as advantageous. Results from examining older immigrants show that those who live alone have higher income and education and are more acculturated. These characteristics may be related to other dimensions of wellbeing, such as more extensive social ties and support because of being more acculturated, and better health, given the well-known socioeconomic status-health gradient [51,52]. Still, we cannot conclude that living alone is the optimal living arrangement for all non-married older immigrants, given study limitations noted above. However, this comparative research provides a reference point for additional research on living alone among older adults, including older immigrants, in other aging societies, particularly where aging immigrants are part of the aging population. The findings also provide useful information for planning and designing age-friendly communities to include older adults who live alone. 

## Figures and Tables

**Table 1:** Descriptive statistics for non-married elderly, Canada and U.S. (in percents).

Characteristic/Variable	Canada			United States		
	All	Canadian-Born	Immigrants	All	U.S.-Born	Immigrants
Live Alone	66.5	70.7	54.8	70.8	73.2	51.7
Demographic Characteristics						
Gender:	100.0	100.0	100.0	100.0	100.0	100.0
Female	69.1	67.8	72.6	68.9	68.2	74.2
Male	30.9	32.2	27.4	31.1	31.8	25.8
Age Groups:	100.0	100.0	100.0	100.0	100.0	100.0
55–59 years old	20.5	21.6	17.4	25.4	25.2	26.9
60–64	15.9	16.5	14.3	19.6	19.6	20.1
65–69	13.4	13.1	14.4	15.5	15.4	16.6
70–74	13.7	13.5	14.1	13	13	13.1
75–79	14.2	13.9	15.1	11.1	11.2	10.1
80–84	12.2	11.4	14.3	8.4	8.5	7.4
85 years old and older	10.1	10.0	10.5	7.0	7.2	5.9
Marital Status:	100.0	100.0	100.0	100.0	100.0	100.0
Divorced	25.5	26.8	22.0	32.2	32.9	26.4
Separated	7.3	7.1	8.1	4.2	3.7	8.1
Widowed	50.0	47.8	56.3	49.2	49.1	50.5
Never-married	17.1	18.4	13.6	14.4	14.3	15.0
Ethnic Origin ^a^:	100.0	100.0	100.0	100.0	100.0	100.0
Single Origin						
Canadian/American ^b^	18.5	24.9	0.6	7.2	8.1	0.2
British	14.0	14.1	13.6	7.4	7.8	3.8
French	6.5	8.4	1.3	0.9	0.9	0.8
Other European	15.3	9.2	32.3	26.2	26.6	23.1
Arab/Middle Eastern	0.7	0.1	2.5	0.5	0.2	3.1
South Asian/Asian Indian	1.7	0.0	6.2	0.3	0.0	2.2
Chinese	2.4	0.1	8.7	0.6	0.1	4.5
Filipino	0.6	0.0	2.2	0.6	0.1	4.6
Korean	0.1	0.0	0.4	0.3	0.0	2.2
Vietnamese	0.2	0.0	0.7	0.2	0.0	2.1
Other Asian	0.3	0.2	0.5	0.6	0.3	2.9
Latin American/Latino/Hispanic	0.3	0.0	1.0	6.6	3.4	32.1
African/Black/Caribbean	1.4	0.1	4.9	11.4	12.0	7.0
Other Single Origin ^c^	1.5	2.0	0.2	13.3	14.3	5.6
Multiple Origins	36.7	40.8	25.1	24.0	26.3	5.9
Economic Characteristics						
Education:	100.0	100.0	100.0	100.0	100.0	100.0
Less than High School	40.5	40.9	39.3	27.2	25.2	43.7
High School Graduate	22.4	22.7	21.5	33.1	34.2	23.8
Post-High School	26.5	26.4	27.1	21.6	22.4	15.0
Bachelor’s Degree	7.5	7.4	7.9	10.3	10.3	10.4
Post-Bachelor’s	3.1	2.7	4.2	7.8	7.9	7.1
Mean Years of Education	11.7	11.5	12.1	12.1	12.3	10.3
Individual Income:	100.0	100.0	100.0	100.0	100.0	100.0
Below $10,000	8.5	7.8	10.3	26.9	24.7	44.4
$10–19,999	37.5	36.5	40.0	29.6	30.2	24.8
$20–39,999	32.5	33.3	30.7	24.9	25.8	17.9
$40–59,999	12.4	13.0	11.0	9.7	10.1	6.4
$60–99,999	6.3	6.5	6.0	5.9	6.2	4.1
$10,000 and over	2.8	2.8	2.9	3.1	3.2	2.4
Mean Individual Income ($)	31,318	31,738	30,143	26,832	27,832	20,755
Homeownership:	100.0	100.0	100.0	100.0	100.0	100.0
Yes	61.5	59.6	66.7	70.2	71.3	61.7
No	38.5	40.4	33.3	29.8	28.7	38.3
Acculturation Characteristics (foreign-born only)						
Duration of Residence in Canada or U.S.:			100.0			100.0
0–9 years			5.5			10.7
10–19			12.6			14.7
20–29			14.4			18.0
30–39			23.8			20.3
40 and more years			43.8			36.4
Mean Years of Duration of Residence			37.7			34.8
Language Proficiency ^d^:			100.0			100.0
(1)			37.6			25.2
(2)			28.6			19.5
(3)			19.1			36.4
(4)			14.6			18.9
Other Characteristics						
Metropolitan Residence:	100.0	100.0	100.0	100.0	100.0	100.0
Yes	65.8	58.5	86.4	75.4	73.1	93.8
No	34.2	41.5	13.6	24.6	26.9	6.2
Sample Size (Number of cases)						
Unweighted	67,948	50,054	17,894	300,573	273,185	27,573
Weighted	2,514,076	1,851,998	662,078	27,821,402	24,721,329	3,100,073

^a^ For Canada, based on responses to the ethnic origin question. For the U.S., based on responses to the ancestry question. Two responses are allowed in the U.S. question while multiple responses are allowed in the Canadian question. ^b^ ‘Canadian’ ethnic origin in Canada, ‘American’ ancestry for the U.S. The latter is recorded if ‘American’ is the only response. ^c^ Includes persons reporting single Aboriginal origin in Canada, and single Native American or Native Alaskan or Native Hawaiian/Other Pacific Islander origin in the U.S. ^d^ The categories are not directly comparable. For Canada, (1) English or French mother tongue or home language; (2) other mother tongue, English or French home language; (3) other mother tongue and home language, knows English or French; (4) other mother tongue and home language, does not know English or French. For the U.S., (1) speaks English only; (2) speaks English very well; (3) speaks English well or not well; (4) does not speak English.

**Table 2:** Model I: observed and predicted (adjusted) percents, living alone, by nativity, non-married elderly, Canada and the U.S. ^a^.

Country/Nativity	Observed	Adjusted
Canada		
Native-born	70.7	68.1
Foreign-born	54.8	64.7
Difference	15.9	3.4
U.S.		
Native-born	73.2	68.9
Foreign-born	51.7	67.3
Difference	21.5	1.6

^a^ Model I was estimated separately for the Canadian and U.S. samples. It includes a dummy variable for nativity. Adjusted or predicted percentages control for age, gender, marital status, individual income, government pension, private retirement income, homeownership, education, ethnic origin, language proficiency, and place of residence. Duration of residence for immigrants was not included because it is collinear with nativity. Predicted probabilities were multiplied by 100 to show the predicted (adjusted) percent living alone.

**Table 3:** Model II: observed and predicted (adjusted) percentages living alone, gender and nativity comparisons, Canada and U.S. ^a^.

Country/Gender/Nativity	Observed	Adjusted
Canada		
Females		
Native-born	70.7	67.2
Foreign-born	52.4	63.5
Difference	18.3	3.7
Males		
Native-born	70.6	70.1
Foreign-born	61.0	67.4
Difference	9.6	2.7
U.S.		
Females		
Native-born	70.6	65.6
Foreign-born	47.3	64.2
Difference	23.3	1.4
Males		
Native-born	78.8	76.0
Foreign-born	64.1	74.1
Difference	14.7	1.9

^a^ Model II was estimated for all non-married elderly, for each of these four groups: Canada/female, Canada/male, U.S./female, U.S./male. It includes a dummy variable for nativity, and all the other explanatory variables included in Model I.

**Table 4:** Model III: predicted probabilities of living alone for categorical explanatory variables, non-married older immigrants in Canada and U.S. ^a^.

Variable Category	Canada		United States	
	Females	Males	Females	Males
A. Demographic Characteristics				
Marital Status:				
Divorced	0.5662	0.6824	0.4957	0.6773
Separated	0.5373	0.7047	0.3972	0.6267
Never-married	0.5496	0.6660	0.4791	0.6690
Widowed	0.5447	0.6460	0.4357	0.5256
B. Economic Factors				
Government Pension Income:				
No	0.5112	0.6361	0.3993	0.5861
Yes	0.5698	0.6872	0.5068	0.6718
Retirement Pension Income:				
No	0.5282	0.6433	0.4453	0.6231
Yes	0.5784	0.7015	0.4893	0.6281
Homeownership:				
No	0.4985	0.6122	0.4254	0.5718
Yes	0.6014	0.7371	0.4913	0.6581
Highest Degree Completed:				
Less than high school	0.5013	0.5812	0.4171	0.5904
High School	0.5462	0.6502	0.4358	0.6025
Post-High School (not Bachelor’s)	0.6054	0.7169	0.5132	0.6606
Bachelor’s Degree	0.6082	0.7229	0.5060	0.6732
Post-Bachelor’s Degree	0.6792	0.7819	0.5569	0.6997
C. Acculturation Factors				
Ethnic Origin:				
Canadian/American	0.5820	0.8103	0.4989	0.8750
British	0.5801	0.6767	0.5944	0.7517
French	0.6303	0.7995	0.6677	0.8484
Other European	0.6347	0.7448	0.6177	0.7529
Arab/Middle Eastern	0.5328	0.7397	0.4855	0.6456
South Asian/Asian Indian	0.3845	0.5316	0.3301	0.5946
Chinese	0.5405	0.6503	0.5232	0.6442
Filipino	0.3272	0.4595	0.3387	0.4851
Korean	0.6881	0.5235	0.5988	0.7636
Vietnamese	0.4147	0.5550	0.4448	0.5352
Other Asian	0.5769	0.7126	0.5035	0.5407
Latin American/Latino/Hispanic	0.4885	0.3765	0.4274	0.6009
African/Black/Caribbean	0.4107	0.5073	0.3816	0.5889
Other Single Origin	0.6173	0.6757	0.6050	0.7609
Multiple Origins	0.4908	0.6122	0.5442	0.6617
Official Language Proficiency ^b^:				
(1)	0.6025	0.7203	0.5279	0.6835
(2)	0.5711	0.6865	0.4549	0.6331
(3)	0.5246	0.5790	0.4611	0.6203
(4)	0.4253	0.5271	0.4079	0.5805
D. Other Controls				
Place of Residence:				
Montreal/Chicago ^c^	0.5127	0.6473	0.4134	0.6130
Toronto/Los Angeles ^c^	0.4983	0.5965	0.4126	0.5686
Vancouver/Miami ^c^	0.5668	0.6772	0.4834	0.6873
---/New York City ^c^	---	---	0.4136	0.5523
---/San Francisco ^c^	---	---	0.4316	0.6086
Other Metropolitan Areas	0.5916	0.7384	0.4737	0.6468
Non-Metropolitan	0.6402	0.7496	0.5193	0.7066

^a^ Model III was estimated for four groups of older immigrants: Canada/female, Canada/male, U.S./female, and U.S./male. ^b^ Language proficiency in official language(s) is not comparable for Canada and the United States. See text and notes for Table 1 for description of how this variable is coded. ^c^ The first city listed is for Canada and the second city is for the U.S.

## Supplementary Materials

The following are available online at https://www.mdpi.com/2227-9032/7/2/68/s1, Table S1A–B: Logistic Regression Analysis Predicting Living Alone for Elderly Adults in Canada and the United States, 2006, Table S2A–D: Logistic Regression Analysis Predicting Living Alone for Elderly Adults in Canada and the United States, by Nativity and Gender, 2006, Table S3A–D: Logistic Regression Analysis Predicting Living Alone for Elderly Immigrants in Canada and the United States, 2006.
